# Ultrasound-Guided Axillary Brachial Plexus Block for the Management of Graft Site Pain During Dressing Change in the Burn-Injured Patient: A Randomized Control Trial

**DOI:** 10.1093/jbcr/irac060

**Published:** 2022-04-29

**Authors:** Cienwen J Town, Haakan Strand, James Johnson, André Van Zundert

**Affiliations:** Department of Anaesthesia and Perioperative Medicine, Royal Brisbane and Women’s Hospital, Queensland, Australia; University of Queensland, School of Nursing, Midwifery and Social Work, Australia; University of Queensland, School of Nursing, Midwifery and Social Work, Australia; Department of Anaesthesia and Perioperative Medicine, Royal Brisbane and Women’s Hospital, Queensland, Australia; Department of Anaesthesia and Perioperative Medicine, Royal Brisbane and Women’s Hospital, Queensland, Australia; University of Queensland Faculty of Medicine & Biomedical Sciences, Australia; University of Queensland Burns, Trauma & Critical Care Research Centre, Australia

## Abstract

Burn injuries requiring split-thickness skin grafting procedures often require ongoing wound aggravation in the form of dressing changes. These dressing changes may cause significant pain due to stimulation of damaged nerve endings in the epidermal layer. A randomized control trial, pilot study, was undertaken to evaluate the impact of ultrasound-guided regional nerve block on the outcome of patient reported pain scores by inpatients requiring dressing changes for hand and upper limb burn injuries. Twenty participants aged >18 years, requiring split-skin grafting for burn injuries of <15% total body surface area were enrolled from a tertiary burns unit between August 2018 and September 2020. Participants were randomized to control (10 participants) or intervention group (10 participants). All participants received analgesia as per their treating team, the intervention group received the addition of an ultrasound-guided axillary brachial plexus block prior to their dressing change procedure. The primary outcome was to assess perceived pain at the graft site as measured by the Numeric Pain Rating Scale (0–10) before, during, and after dressing change procedure. There was strong evidence of a difference in the adjusted mean change score between groups, with a mean reduction of 4.3 in the intervention group, indicating reduced pain, and a mean increase of 1.2 in the control group (*P* < .001). No adverse events occurred in either group, and the addition of ultrasound-guided regional anesthesia (RA) for the treatment of dressing pain was determined to be a safe and effective intervention.

Burn injuries vary in size, depth, severity, and cause. Superficial burns are managed with appropriate topical treatments and dressings, while deeper partial, mixed, and full thickness burns may require surgical debridement and skin grafting.^1^

The experience of burn pain is predominantly caused by damage or disruption to cutaneous nerve endings, resulting in changes to perceived temperature, touch, and pain.^1^ Neuropathic pain is notoriously difficult to treat and has the potential to result in long-term chronic pain development.^2^ For patients who undergo split-thickness skin grafting, significant pain is often experienced in the donor site; which is typically located on the anterior thigh.^3–6^ Burn injuries that require debridement and skin grafting will require ongoing wound aggravation in the form of repeated debridement, dressing changes, and physical therapy. These interventions have the potential to cause pain, significant discomfort and distress to patients.^2^

Several studies have looked at the addition of RA for the management of donor site pain in the burn-injured patient.^3,4,6–10^ RA is a method of providing analgesia or anesthesia to a targeted area of the body via sensory blockade. Ultrasound-guided RA is a technique that provides safe, effective deposition of local anesthetic (LA) to a targeted nerve, or bundle of nerves under ultrasound guidance.^3^ The generalized outcome of the published studies looking at nerve blocks and burns patients have demonstrated that RA provides a safe and effective addition to the multimodal management of donor and graft site pain. None of the published studies evaluate RA as an intervention for graft site pain during dressing change.

Early multimodal pain relief is imperative for management of acute pain in the burn-injured patient. Patients are at increased risk of developing long-term chronic pain and psychiatric trauma if acute pain is poorly managed.^11^ RA is an effective primary pain management technique or adjunct to a multimodal regime. However, its application in burns is still underreported and underutilized.^5^ In particular, an area of burn treatment that has yet to be explored in partnership with RA is that of dressing changes and postoperative procedural pain. Dressing changes typically occur 3 to 5 days post the split-skin grafting procedure and can involve removal of staples, minor debridement, and redressing or splinting. These interventions are well known to cause pain for patients and typically opioids are used alone or in conjunction with other analgesics and sedatives depending on the TBSA of the burn, and the existing pain tolerance of the patient.

This paper reports on a randomized control trial that utilized ultrasound-guided regional nerve blocks for the management of burn-related procedural pain in the upper limbs. The axillary brachial plexus block (ABPB) targets the three terminal branches of the brachial plexus: the median, the ulnar, and the radial nerve.^12^ The ABPB block reliably blocks sensation to the forearm hand and wrist. Regional nerve blocks may be administered as a single injection, or the insertion of a catheter to provide continuous or intermittent bolus LA. Single injection nerve blocks typically provide analgesia for between 3 and 12 hours depending on the type of LA used; while catheters remain in situ for several days.

Skin grafting procedures are the most common surgical treatment for burn injuries. In the case of burn injuries that occur to mobile areas of the body, splinting is required for 3 to 5 days to immobilize the grafted area, preserve the skin graft and encourage adhesion. Small grafted areas of the fingers and hands may require a significant number of staples to secure the graft, consequently, removal of staples at the initial dressing change and the immediate range of motion (ROM) exercises may cause significant discomfort.

The intention of this study is to evaluate the efficacy of RA as a pain-relieving intervention in patients who require postoperative dressing changes to upper limb burn injuries. These initial dressing changes take place in a ward setting, 3 to 5 days post skin grafting procedure.

## METHODS

Recruitment for this two-group parallel randomized control trial occurred between August 2018 and September 2020, at a tertiary hospital acute burn unit. Ethical approval was granted by an independent Human Research Ethics Committee (HREC/17/QRBW/54). Patients who were scheduled for skin grafting procedures for upper limb burn injuries and subsequently required change of dressing as an inpatient postoperatively, were approached. Additional inclusion criteria were <15% TBSA burns, fluency in written and spoken English and ability to provide informed consent, anticipated length of hospital stay of >24 hours postsurgery. Exclusion criteria were intubation, vascular disease or active infection, and contraindication to RA. Twenty participants, aged 18 years or more, were approached and consented to participate in the study. Simple randomization was conducted by a third party, closed opaque enveloped method, with 10 participants randomized, respectively, to the intervention group or the control group.

All 20 participants had a standard analgesia regime as per the discretion of the treating team and anesthetist during their initial dressing change and removal of staples post skin grafting procedure. The 10 participants in the intervention group received an ultrasound-guided ABPB of 0.375% ropivacaine 20 to 30 ml prior to their dressing change procedure. Block administration occurred at the patient bedside, or in the anesthetic “Green Room”; a theater induction bay reserved for regional procedures and staffed with an anesthetic consultant and registrar each day. Participants in the intervention group received supplemental oxygen and routine observation monitoring, including noninvasive blood pressure and pulse oximetry during the ABPB administration. Standardized monitoring in conjunction with ultrasound guidance aims to reduce the risks of: nerve injury, inadvertent damage to adjacent structures, and LA toxicity. Participants received 1 to 4 mg intravenous midazolam as sedation as required. The ultrasound-guided ABPB was administered within 1 to 2 hours prior to dressing change by qualified anesthetists, employing standard aseptic technique. The primary outcome of the study was to assess pain at the graft site during dressing change, as measured by the Numeric Pain Rating Scale 0–10 (NPRS). This pain score was then compared to the baseline predressing NPRS scores in both the control and intervention groups.

Further clinical outcomes of interest included, pain score at the donor site during dressing change, requirement for analgesia and pregabalin dosages, nausea, and vomiting, and achievement of physical therapy ROM goals. Pain management during dressing change was left to the discretion of the Burn team. Morphine equivalent opioid conversion provides a comparison for pharmaceutical opioid dosing; furthermore, it allows for comparison of multiple pharmaceutical opioids that have similar analgesic effects.^13–17^ Pain scores at the donor site during dressing change procedures were collected for comparison and understanding of background pain requirements and site-specific discomfort.

Data were summarized with frequencies and percentage for categorical data, median (interquartile range [IQR]) for non-normally distributed continuous data and mean (*SD*) for normally distributed continuous data. The change in skin graft pain from predressing to dressing change was analyzed using the ANCOVA of NPRS change, adjusted for pre-NPRS scores between the control and intervention groups. Associations between predressing NPRS scores and groups were assessed using Mann–Whitney *U*-tests. Statistical significance was set at a *P* value <.05 (two sided). STATA 15 (Stat Corp LLC) was used for all analyses and figure produced in R statistical package version 4.0.2.

## RESULTS

Demographic data were collected for comparison and better understanding of background pain. Characteristics were similar across both the intervention and control group as seen in Table 1.

**Table 1.** Patient characteristics for control and nerve block intervention groups

Characteristic	Overall	Control	Intervention
	n = 20	n = 10	n = 10
Age (yr), mean (*SD*)	36.3 (14.7)	34.8 (15.4)	37.9 (14.7)
Gender, n (%)			
Female	1 (5%)	0 (0%)	1 (10%)
Male	19 (95%)	10 (100%)	9 (90%)
BMI, median (IQR)	26.4 (24.5–31.4)	26.1 (22.4–30.0)	27.1 (25.9–32.9)
ASA, n (%)			
1	6 (30%)	3 (30%)	3 (30%)
2	12 (60%)	6 (60%)	6 (60%)
3	2 (10%)	1 (10%)	1 (10%)
TBSA, n (%)			
<2%	5 (25%)	4 (40%)	1 (10%)
2%–4.9%	8 (40%)	3 (30%)	5 (50%)
≥5%	7 (35%)	3 (30%)	4 (40%)
Burn depth, n (%)			
Superficial and partial thickness	6 (30%)	4 (40%)	2 (20%)
Partial thickness	4 (20%)	4 (40%)	0 (0%)
Partial and full thickness	9 (45%)	2 (20%)	7 (70%)
Full thickness	1 (5%)	0 (0%)	1 (10%)
Burn type, n (%)			
Flame	9 (45%)	6 (60%)	3 (30%)
Hot oil/liquid	10 (50%)	4 (40%)	6 (60%)
Electrical	1 (5%)	0 (0%)	1 (10%)

*ASA*, American Society of Anesthesiology Physical Status; *BMI*, body mass index; *IQR*, interquartile range; *NPRS*, Numeric Pain Rating Scale; *PACU*, Post Anaesthetic Care Unit.

**Figure 1.**
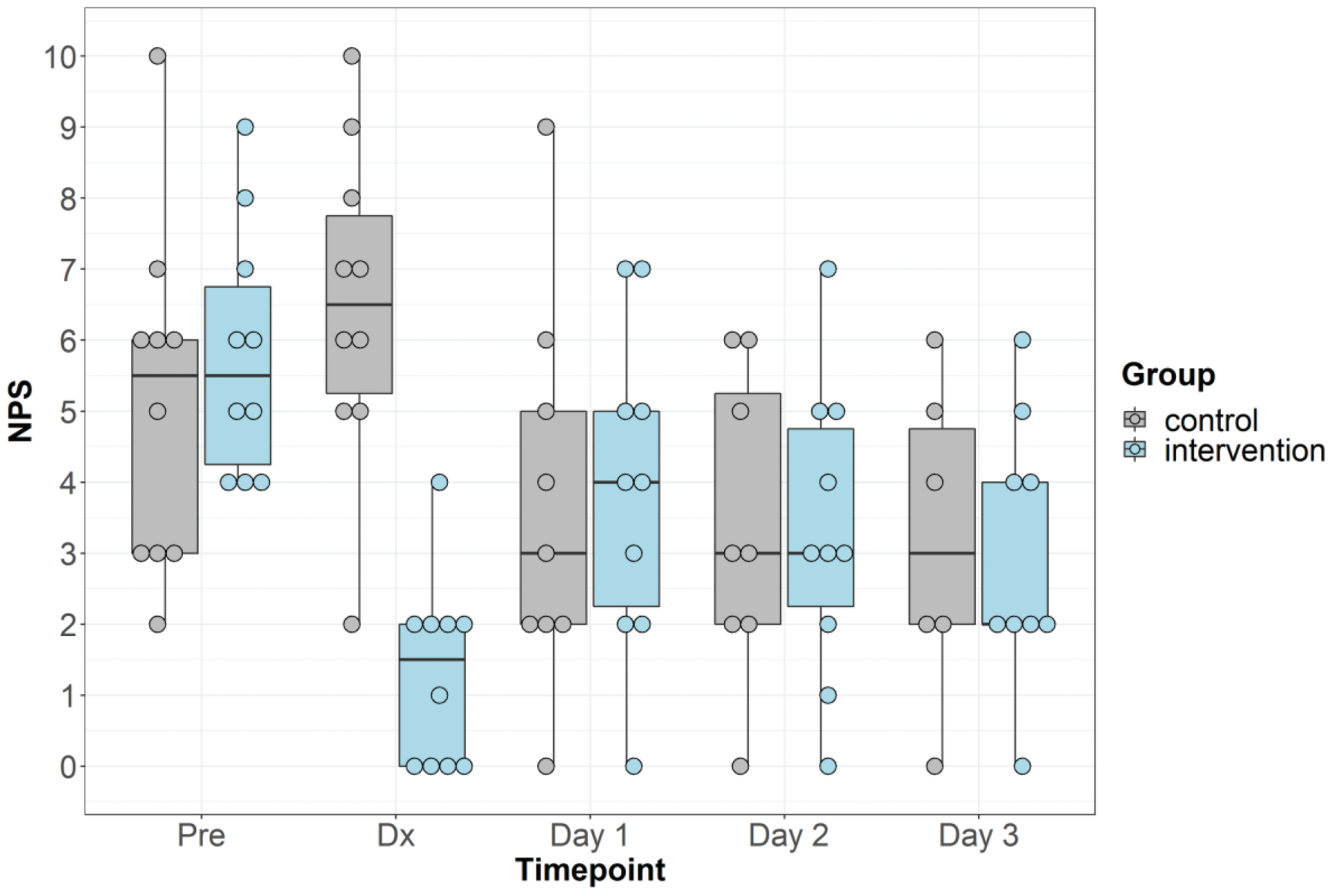
Graft site pain comparison between the control and block intervention groups pre-dressing, at dressing change (dx) and over 3 follow-up days.

The primary outcome was to assess pain at the graft site as measured by the NPRS and compare it to predressing NPRS scores in both the control and intervention group. Table 2 displays the results of the primary outcome ANCOVA for comparing the predressing pain adjusted change in NPRS at the time of dressing change between control and intervention groups. For NPRS, moving closer to zero is indicative of a reduction in pain (negative change score). There was very strong evidence of a difference in the adjusted mean change of pain score between groups, with a mean change of −4.3 in the intervention group, indicating reduced pain, and a mean change of 1.2 in the control group, indicating increased pain (*F*(1,17) = 47.1, *P* < .001). There was no significant difference in predressing pain scores between groups (*P* = .47). Figure 1 displays a comparison of the control and intervention groups pain scores over all time points assessed.

**Table 2.** Comparison between predressing pain adjusted change in NPRS for control and intervention groups at the time of dressing change

	Control	Intervention	*P*
	n = 10	n = 10	
Pre-NPRS, mean (*SD*)	5.1 (2.4)	5.8 (1.8)	.47
Raw change in NPRS, mean (95% CI)	1.40 (−0.12 to 2.92)	−4.50 (−6.09 to −2.91)	–
Adjusted change in NPRS, mean (95% CI)	1.18 (0.00 to 2.35)	−4.28 (−5.45 to −3.10)	<.001*

*NPRS*, Numeric Pain Rating Scale.

*ANCOVA *F*(1,10) = 36.8.

The median duration of the sensory nerve block in the intervention group was 11.0 hours (IQR 9.2–11.3). There were no adverse reactions or nerve injuries within the intervention group, and 50% received the addition of midazolam as a preprocedural medication. There were no failed block attempts, or any LA top ups required for incomplete block. Pain relief administration during dressing change was left to the discretion of the treating team. Median (IQR) NPRS in donor site during dressing change was 6.0 (3.0–10.0) for the control group and 5.5 (4.0–8.0) for the intervention group. Donor site pain scores were recorded at time of dressing change, and daily for 3 days postdressing change with no statistically significant differences between the groups. During dressing change, 40% of control participants required Nitrous oxide, and neither group reported any nausea, vomiting, or increased sedation during dressing change. Pregabalin administration during dressing change was similar across groups with 70% of the control group, and 80% of the intervention group receiving a median dose of 150 mg.

Table 3 provides the median (IQR) morphine equivalent comparison between control and intervention groups at the following time points: predressing change, during dressing change, and day 1 to 3 postdressing change procedure. The median morphine equivalent dose is higher in the intervention group across all time points; however, the difference was not statistically significant.

**Table 3.** Patient morphine equivalents between groups (using Kruskal–Wallis test)

Median (IQR) Morph Equivalent	Total	Control	Intervention	*P*
	N = 20	N = 10	N = 10	
Predressing	27.0 (11.3–68.8)	23.3 (7.5–55.5)	41.5 (15.0–80.0)	.36
Dressing change	33.3 (18.8–62.3)	33.3 (15.0–60.0)	41.3 (22.5–65.0)	.79
Day 1 (n = 19)	16.0 (8.0–54.0)	15.0 (7.5–38.0)	30.0 (15.0–54.0)	.39
Day 2 (n = 18)	18.8 (7.5–54.0)	7.8 (7.5–30.5)	45.0 (15.0–67.5)	.12
Day 3 (n = 15)	30.0 (8.0–60.0)	4.0 (0.0–30.0)	45.0 (30.0–60.0)	.057

*IQR*, interquartile range.

The average length of stay was 8.7 days for the control group and 12.1 days for the intervention group. Sixty percent of participants in each group were discharged with an opioid script, it is unknown if the scripts were dispensed.

## DISCUSSION

The results of this study demonstrate the effectiveness of regional nerve blocks for the management of procedural pain during upper limb dressing changes in the burn-injured patient. RA is currently underutilized as an analgesic intervention in the burn-injured patient. The contraindications in this cohort are few, and the potential benefits are many.^5,11^ To date there are no published clinical trials focusing on regional nerve blocks during burn dressing changes, this is likely due to a number of reasons including: perceived infection risk; poor access to experienced anesthetic clinicians and procedural equipment; focus on chronic pain interventions; and the paucity of published literature to guide a change in practice.

Regional nerve blocks are not appropriate for every burn-injured patient during dressing change, and there are limiting factors including appropriate patient selection, and suitable body region of the burn amenable to an analgesic nerve block procedure. Hand and upper limb burns were chosen for this study due to the large number of staples required to anchor grafts, and due to the early hand mobilization required to ensure optimum rehabilitation. All 10 patients who received the ABPB were unable to perform active ROM for the physiotherapy session immediately postdressing change due to motor block. However, passive ROM was achieved in all 10 patients without difficulty and there were no limitations to this group achieving their targeted active ROM on day 1 post the regional block. Motor block is a known side effect of RA blocks, and participants were made aware of what to expect regarding a lack of sensation and movement. Participants in the intervention group had their hands/arms placed in slings where required, to protect them from accidental injury while mobilizing. The median sensory block length was 11.0 hours and none of the intervention group sustained any nerve injuries.

Morphine equivalent pain relief consumption was higher in the intervention group across all time points from predressing change until day 3 postdressing change. This could be due to a number of factors, starting with the slightly larger TBSA% of the intervention group, with 40% having burns ≥5% TBSA and 50% having burns 2% to 4.9% TBSA; compared to only 20% and 30%, respectively, in the control group. This larger area of burn injury could explain the higher requirement for pain relief to treat areas of burn outside the region covered by the ABPB. As per Table 3, there was only a 0.2 mg morphine equivalent increase between the predressing and during dressing change requirements for the intervention group, compared to a 10 mg increase in the control group. While the intervention group required higher morphine equivalent doses overall, their background pain scores were comparable and the increase in opioid requirement during dressing change is clinically representative of the increase in pain experienced by the control group. Not included in the morphine equivalent dosing was the use of nitrous oxide in the control group; 40% of control participants required nitrous oxide compared to 0% of the intervention group. Nitrous oxide was not withheld from any participants; it was provided as per the treating team and patient requirements.

Ultrasound guidance has introduced an added level of precision and safety to RA through the ability to sight major anatomical structures and nerves; this visual guidance makes nerve or vascular injury less likely.^12^ Careful aspiration prior to LA injection decreases intravascular injection. The risk of LA toxicity is low due to weight-based dosing and exclusion of patients with contraindications to LA.^18^ The median age was 34.5 years with 90% of participants receiving an American Society of Anaesthesiology (ASA) physical status classification score of ≤2, making them a group of participants with mild or no systemic disease. None of the participants reported preexisting chronic pain conditions, or opioid dependence. Ultrasound-guided regional nerve blocks have been growing in popularity throughout the last three decades, and their application to the burn-injured population should not be overlooked.^19^

Successful implementation of regional blocks for burn injury procedural pain relies on the availability of trained anesthetists and appropriate monitoring equipment. This study was possible due to the staffing of one consultant anesthetist and one anesthetic registrar every day for the purpose of administering regional anesthetic blocks to surgical patients within the hospital. This resource is invaluable to patients who receive the added anesthesia or analgesia; and the anesthetic registrars who gain education administering a variety of regional nerve blocks under supervision. This resource makes administering regional nerve blocks convenient, safe, and timely. The administration of regional nerve blocks for dressing change may prevent some patients from having an additional general anesthetic. Providing general anesthesia for dressing changes requires significantly more hospital resources and additional risk and recovery time for a patient.

There are limitations to the use of regional anesthetic blocks in burns patients, those include: patients with large TBSA burns, or burns in locations that cannot be covered with RA; burn wounds that will require frequent and ongoing wound aggravation; patients with active infection; and patients who are not appropriate for RA due to comorbidities, allergy to LA, or altered anatomy.

The short duration of dressing changes is perhaps one contributing factor to the under-treatment of pain during procedures; there may be a tendency to under-treat procedural pain due to the perception that it should be tolerable for a short period. However, pain associated with dressing changes is acute pain and can be severe. It comes with the same potential adverse consequences as acute pain occurring in the perioperative setting. Poorly managed acute pain can lead to; impaired sleep, delayed recovery, risk of chronic pain development, and reduced quality of life. Another factor is the opioid-based approach to treating burn pain. Opioids do not target the neuropathic pain specific to burns and may often result in side effects like constipation and sedation.^2^

This study does present limitations, the study size is small and larger, multicenter randomized control trials would be beneficial. Further studies evaluating different regional blocks for dressing and procedural changes would be of interest. Recruitment for this trial was prolonged due to the emergence of a change in practice. Burns consultants began booking patients for scheduled nerve blocks for dressing changes, making patients ineligible for study recruitment. This was a positive reflection on the perceived benefits of RA for pain relief, however, it did delay recruitment.

Participants in the intervention group were asked prior to discharge if they would opt to receive a regional nerve block in the future and 100% of participants said yes.

## CONCLUSION

RA should be considered as a pain relief option for the management of procedural pain in the burn-injured patient. Ultrasound-guided regional nerve blocks have the potential to significantly lower pain scores and provide a safe and effective addition to multimodal pain relief in burn-injured patients.
