# Correction to: Ultrasound-Guided Axillary Brachial Plexus Block for the Management of Graft Site Pain During Dressing Change in the Burn-Injured Patient: A Randomized Control Trial

**DOI:** 10.1093/jbcr/irac150

**Published:** 2022-10-15

This is a correction to: Cienwen J Town, GradCertEd, BN, Haakan Strand, PhD, James Johnson, FANZCA, MBBS, B.Applied Science, André Van Zundert, MD, PhD, FRCA, EDRA, FANZCA, Ultrasound-Guided Axillary Brachial Plexus Block for the Management of Graft Site Pain During Dressing Change in the Burn-Injured Patient: A Randomized Control Trial, *Journal of Burn Care & Research*, 2022, irac060, https://doi.org/10.1093/jbcr/irac060

In the originally published version of this manuscript, one of the sentences in the Results section read: “There was very strong evidence of a difference in the adjusted mean change of pain score between groups, with a mean change of −4.3 in the intervention group, indicating reduced pain, and a mean change of 1.2 in the control group, indicating increased pain (*F*(1,10) = 36.8, *P* <.001).” It was replaced with “There was very strong evidence of a difference in the adjusted mean change of pain score between groups, with a mean change of −4.3 in the intervention group, indicating reduced pain, and a mean change of 1.2 in the control group, indicating increased pain (*F*(1,17) = 47.1, *P* <.001)

The following sentence was changed from “There was no significant difference in predressing pain scores between groups (*P* =.42)” to “There was no significant difference in predressing pain scores between groups (*P* =.47).”

In Table 2, two values were updated:

Adjusted change to NPRS, mean (95% CI) Control was changed from 1.16 (0.04 to 2.37) to 1.18 (0.00 to 2.35) and Intervention was changed from −4.26 (−5.47 to −3.05) to −4.28 (−5.45 to −3.10).

Acknowledgement section was updated to say “Acknowledgment to Stacey Llewellyn for statistical analysis and Dr Georgia Taylor for contribution to data collection” instead of “Acknowledgement to Dr Georgia Taylor for her contribution to data collection.”

These errors have been corrected.

